# Ferroptosis Mediates Pulmonary Fibrosis: Implications for the Effect of *Astragalus* and *Panax notoginseng* Decoction

**DOI:** 10.1155/2024/5554886

**Published:** 2024-03-29

**Authors:** Jing Wen, Cui Wang, Li-yun Song, Yin-ying Wang, Peng-tao Liang, Wen-lin Pang, Wen Yin, Qiang Zhang, Wei-tian Zhao, Xue-ping Sun, Jin-yuan Yan, Zhong-shan Yang

**Affiliations:** ^1^Yunnan Provincial Key Laboratory of Integrated Traditional Chinese and Western Medicine for Chronic Disease in Prevention and Treatment, Yunnan University of Chinese Medicine, Kunming, Yunnan, China; ^2^Dali Prefectural Hospital of Traditional Chinese Medicine, Dali, Yunnan, China; ^3^Central Laboratory, Kunming Medical University Second Hospital, Kunming, Yunnan, China

## Abstract

**Objective:**

To investigate the mechanism through which *Astragalus* and *Panax notoginseng* decoction (APD) facilitates the treatment of ferroptosis-mediated pulmonary fibrosis.

**Materials and Methods:**

First, the electromedical measurement systems were used to measure respiratory function in mice; the lungs were then collected for histological staining. Potential pharmacologic targets were predicted via network pharmacology. Finally, tests including immunohistochemistry, reverse transcription-quantitative polymerase chain reaction, and western blotting were used to evaluate the relative expression levels of collagen, transforming growth factor *β*, *α*-smooth muscle actin, hydroxyproline, and ferroptosis-related genes (GPX4, SLC7A11, ACSL4, and PTGS2) and candidates involved in the mediation of pathways associated with ferroptosis (Hif-1*α* and EGFR).

**Results:**

APD prevented the occurrence of restrictive ventilation dysfunction induced by ferroptosis. Extracellular matrix and collagen fiber deposition were significantly reduced when the APD group compared with the model group; furthermore, ferroptosis was attenuated, expression of PTGS2 and ACSL4 increased, and expression of GPX4 and SLC7A11 decreased. In the APD group, the candidates related to the mediation of ferroptosis (Hif-1*α* and EGFR) decreased compared with the model group. *Discussion and Conclusions*. APD may ameliorate restrictive ventilatory dysfunction through the inhibition of ferroptosis. This was achieved through the attenuation of collagen deposition and inflammatory recruitment in pulmonary fibrosis. The underlying mechanisms might involve Hif-1*α* and EGFR.

## 1. Introduction

The final common endpoint of several acute and chronic lung diseases is pulmonary fibrosis, which is pathologically characterized by the abnormal infiltration of inflammatory cells in the interstitium of the lung and excessive deposition of extracellular matrix in the lung parenchyma [1, 2]. Typically, pulmonary fibrosis presents with symptoms such as progressive dyspnea and restrictive respiratory dysfunction [3]. Most patients who experience respiratory failure develop restrictive ventilatory dysfunction within 2–3 years after diagnosis of idiopathic pulmonary fibrosis, and the majority of these patients eventually die [4]. However, in addition to the lack of clarity on the causes and mechanisms of pulmonary fibrosis, there is a paucity of effective treatments. Therefore, elucidation of the pathogenesis and progression of pulmonary fibrosis is urgently needed for the development of effective treatments and treatment strategies.

Ferroptosis—a form of regulatory cell death, is characterized by the accumulation of iron-dependent lipid peroxides [5]. Oxidative stress and iron accumulation play essential roles in ferroptosis [6]. Initially, abnormalities in glutathione metabolism were considered the only ferroptosis-associated mechanistic pathway. Glutathione peroxidase 4 (GPX4) and solute carrier family 7 member 11 (SLC7A11) reduce the intracellular antioxidant capacity and are considered crucial mediators of ferroptosis [7]. Mitochondria are reported to shrink and deform during ferroptosis [8]. Cyclooxygenase (COX) catalyzes the synthesis of the prostaglandin family through the action of arachidonic acid (AA) in vivo. In mammalian cells, COX is expressed in two subtypes as follows: COX-1 and COX-2. COX-2 is an inducible enzyme encoded by the prostaglandin-endoperoxide synthase 2 (PTGS2) gene, and its function involves the metabolization of AA to prostaglandin. Yang et al. treated 83 oxidative stress-related genes with erastin or RSL3—a ferroptosis agonist. Among these genes, PTGS2 was the most upregulated [9]. acyl-CoA synthetase long-chain family member 4 (ACSL4) is an isoenzyme that preferentially catalyzes several polyunsaturated fatty acids and is involved in the transformation of AA into AA-CoA. This consequently promotes ferroptosis [10]. Owing to the abovementioned reasons, PTGS2 and ACSL4 are therefore considered essential genes for the identification of ferroptosis.

Several lines of evidence suggested that ferroptosis is elevated in *vitro* and *vivo* experiments' reports with pulmonary fibrosis. Cheng et al. observed substantial iron deposition in the lungs of patients with pulmonary fibrosis and noted that mice and alveolar type II (ATII) cells treated with bleomycin (BLM) showed iron deposition. Furthermore, the authors observed decreased iron amine iron deposition and ferroptosis in ATII cells, which ameliorated the fibrotic effects of BLM [11]. Recent studies reported the occurrence of lipid peroxidation, glutathione depletion, and reduction of SLC7A11 (the upstream molecule formed by Gpx4) in A549 cells stimulated by transforming growth factor beta-1 (TGF-*β*1) to stimulate epithelial-mesenchymal transition [12]. Despite the abovementioned observation, information on the mechanisms that regulate ferroptosis in pulmonary fibrosis is limited.

Recent studies have demonstrated significant antifibrotic effects of *Astragalus* and *Panax notoginseng*. Total flavonoids from Astragalus membranaceus effectively improve pulmonary fibrosis by modulating inflammatory responses and promoting epithelial cell regeneration [13]. In addition, Astragalus polysaccharides aid in alleviating bleomycin-induced pulmonary fibrosis through the inhibition of the TLR4/NF-*κ*B signaling pathway and modulation of gut microbiota [14]. On the other hand, active components of *Panax notoginseng*, such as notoginsenoside R1, show inhibition of hepatic stellate cell activation, with significant antioxidant and anti-inflammatory properties, substantially reducing liver fibrosis induced by carbon tetrachloride [15]. Furthermore, polysaccharides in *Panax notoginseng* inhibit the progression of TGF-*β*-induced epithelial-mesenchymal transition (EMT) and the expression of fibrosis-related proteins in human renal tubular cells [16]. These findings underscore the importance of components in Astragalus membranaceus and Panax notoginseng in inhibiting the process of fibrosis. *Astragalus*, *Panax notoginseng*, and their constituent monomers show antifibrotic effects [17, 18]. In a previous study, we reported a formulation that alleviated pulmonary fibrosis in mice [19]. In our prior study, we discovered that astragaloside IV's efficacy in reversing extracellular matrix deposition and inflammation in mice with pulmonary fibrosis may be attributed to the activation of autophagic flux and autophagic vacuoles [20].

In the present study, we aimed to explore the role of ferroptosis in pulmonary fibrosis in mice; furthermore, we aimed to elucidate the underlying mechanisms and the ferroptosis targets of *Astragalus* and *Panax notoginseng* decoction (APD).

## 2. Materials and Methods

### 2.1. Animals

A total of 30 specific pathogen-free (SPF) C57BL/6 mice (mean body weight, 22 ± 2 g, male) were purchased from Chengdu Dashuo Co., Ltd., with the animal license number SCXK [21] 2020-030. These mice were raised under general conditions at the Animal Center of Yunnan University of Traditional Chinese Medicine. On the first day of the experiment, a model of pulmonary fibrosis was replicated by a single tracheal instillation of bleomycin (Hisun Ruihui Pharmaceutical Co., Ltd., Zhejiang, China) at a dose of 5 mg·kg^−1^. Seven days later, the mice were randomly divided into three groups: the model group, the APD treatment group, and the control group. The model group received the same tracheal instillation of bleomycin without further treatment. The APD group was treated with Astragalus polysaccharide derivative (APD) through oral gavage, starting from day 8 postbleomycin administration, once daily for a total of 21 days. The control group received an identical volume of normal saline via tracheal instillation and was administered saline gavage in a similar manner to the APD group.

The experiments were approved by the Laboratory Animal Welfare and Ethics Committee of Yunnan University of Chinese Medicine (Approval No. R-06202079, Yunnan, China). All animal care and experimental procedures adhered strictly to the guidelines of Chinese Laboratory Animals' Welfare and Ethics.

### 2.2. Preparation and LC-MS/MS Analysis of APD

APD was prepared using a human-to-mouse dose conversion based on the body surface area [21]. The decoction included 30 g of Astragalus (Guangdong Huiqun Traditional Chinese Medicine Co., Ltd.) and 9 g of Panax notoginseng (Yunnan Xianghui Pharmaceutical Co., Ltd.). The mixture, with 300 mL of water, was decocted for 30 minutes and concentrated to 0.5 g crude drug/mL. Mice were administered 0.2 mL/day via gavage. A 2 mL sample was analyzed using LC-MS/MS at Shanghai Biotree Biomedical Technology Co., Ltd., employing an ultra-high-performance liquid chromatography (UHPLC) system (Vanquish, Thermo Fisher Scientific) with a UPLC BEH C18 column. The mobile phase consisted of 0.1% formic acid in water and acetonitrile, with a specified elution gradient. MS and MS/MS data were acquired using an Orbitrap Exploris 120 mass spectrometer and Xcalibur software in IDA mode. The settings included a mass range of 100–1500, a sheath gas flow rate of 30 Arb, an aux gas flow rate of 10 Arb, an ion transfer tube temperature of 350°C, a vaporizer temperature of 350°C, a full MS resolution of 60000, an MS/MS resolution of 15000, a collision energy in NCE mode of 16/38/42, and a spray voltage of +5.5 kV (positive) or −4 kV (negative).

### 2.3. Respiratory Function Assessment Using the Electromedical System

Mice were anesthetized, and a tracheotomy was performed for intubation. Respiratory parameters including static pulmonary compliance (Cchord), total lung capacity (TLC), inspiratory capacity (IC), functional residual capacity (FRC), and forced vital capacity (FVC) were measured using the electromedical measurement system (Information Display Systems Ltd.).

### 2.4. Hydroxyproline Content Measurement

An appropriate amount of lung tissue was weighed, and a 10% tissue homogenate was prepared. The hydroxyproline content was measured according to the manufacturer's instructions.

### 2.5. Histopathology

The lung tissue was fixed and dehydrated with paraformaldehyde fixative, embedded in paraffin, and sliced into paraffin slices. Hematoxylin and eosin staining and Masson staining were performed. The structure, inflammation, and collagen accumulation levels of the lung tissue in each group were observed and compared *via* optical microscopy.

### 2.6. EGFR Immunohistochemistry Protocol

Paraffin sections were dehydrated and underwent antigen retrieval in citric acid buffer. Endogenous peroxidase activity was blocked with 3% hydrogen peroxide, followed by serum blocking. Sections were then incubated with anti-EGFR rabbit pAb (Servicebio, GB111083) and corresponding secondary antibodies. DAB staining highlighted positive areas, with hematoxylin counterstaining for nuclei. Sections were dehydrated and mounted for microscopic analysis. The positive area ratio was quantified using Aipathwell software [22].

### 2.7. Immunofluorescence for Lung ROS Detection

Frozen lung tissue sections were thawed and treated with a liquid blocker pen. After quenching spontaneous fluorescence, ROS staining (D7008, Sigma) was applied and incubated in the dark. Sections were washed and incubated with DAPI (G1012, Servicebio). Postwashing, sections were mounted and examined under a fluorescent microscope. Nuclei appeared blue (DAPI), and ROS-positive cells appeared fluoresced red.

### 2.8. Observation of Mitochondrial Morphology via Transmission Electron Microscopy

Lung samples were fixed in precooled electron microscope fixative (G1102, Servicebio) and postfixed in 1% osmic acid. After room temperature dehydration and infiltration, samples were embedded and polymerized at 60°C for 48 hours. Ultrathin sections (60–80 nm) were prepared, placed on copper meshes, stained, and observed under a transmission electron microscope (HT7800/HT7700, Hitachi) for mitochondrial morphology analysis.

### 2.9. Reverse Transcription-Quantitative Polymerase Chain Reaction (RT-qPCR)

We utilized TRIzol (Takara, Dalian, China) to extract mRNA from lung tissue; the mRNA samples were reverse transcribed into cDNA and amplified, and the relative expression of target gene mRNA was calculated according to 2^−ΔΔCT^. The sequence of primers has been presented in Table 1.

Reagents had been obtained from Hifair III 1st Strand cDNA Synthesis SuperMix for qPCR (gDNA digester plus) (11141ES60, Yisheng Biotech (Shanghai) Co., Ltd.) and Hieff qPCR SYBR Green Master Mix (Low Rox Plus) (11202ES08, Yisheng Biotech (Shanghai) Co., Ltd.).

### 2.10. Western Blotting

Lung tissue was triturated with lysis buffer and steel beads, followed by centrifugation to extract the supernatant. Protein concentration was determined using an enhanced BCA protein assay kit (Beyotime, B10010). After plotting the standard curve and calculating protein amounts, the supernatant was mixed with loading buffer, boiled, and stored at −80°C. SDS-PAGE transferred proteins to a gel, which was then transferred to a membrane for overnight incubation with primary antibodies: anti-TGF-*β*1 (ab179695, Abcam), *α*-SMA (AF1032, Affinity), Hif-1A (BF8002, Affinity), and anti-*β*-actin (BD Bioscience, 521796). Post-secondary antibody application, the membrane was washed and imaged using GeneGnome XRQ Chemiluminescence Imaging System (Syngene, SYGNO104187). Image J quantified the grayscale levels.

### 2.11. Network Pharmacology of APD Targets in Pulmonary Fibrosis and Ferroptosis

To identify the targets of APD, ferroptosis, and pulmonary fibrosis, the TCMSP database (https://tcmsp-e.com/tcmsp.php) was queried using oral bioavailability (OB) ≥30% and drug-likeness (DL) ≥0.18 as criteria. Active components were identified, and target proteins were rectified using UniProt. Disease genes related to ferroptosis and pulmonary fibrosis were searched in DisGeNET (https://www.disgenet.org/), GeneCards (https://www.genecards.org/), and OMIM (https://omim.org/). These predicted target genes were combined to form disease-related gene data. Venny2.1 (https://bioinfogp.cnb.csic.es/tools/venny/index.html) was utilized to determine common targets and create Venn diagrams.

### 2.12. Statistical Analysis

GraphPad Prism 8 (GraphPad Software) was used for plotting and data analysis. Measurement data were expressed as mean ± standard deviation. A significant difference was determined by the one-way ANOVA test followed by the Student–Newman–Keuls test. *P* < 0.05 indicated statistical significance.

## 3. Results

### 3.1. APD Alleviates Disease Symptoms in Mice with Pulmonary Fibrosis

**Figure 1:**
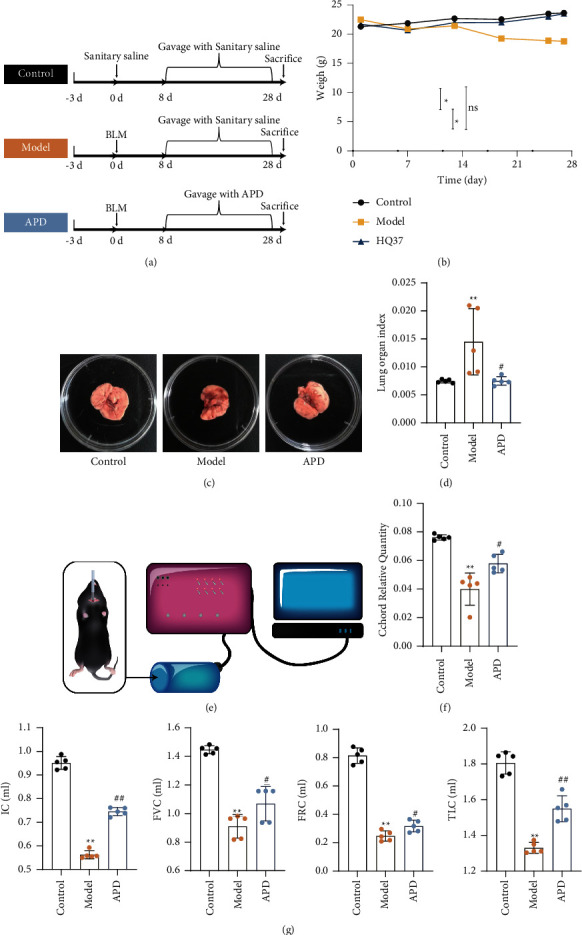
APD enhances recovery in pulmonary fibrosis mice: body weight, respiratory function, and lung index improvements. (a) Timeline diagram of experimental design (^*∗*^*P* < 0.05, ns indicates no significant difference). (b) The average weight change of each group of mice (*n* = 5). (c) On the 28^th^ day, the lungs were removed to observe tissue morphology. (d) Changes in the lung organ index of mice in each group (*n* = 5). (e–g) Cchord, IC, FVC, FRC, and TLC status of pulmonary fibrosis mice (*n* = 5). The data were obtained through three independent experiments. ^*∗*^*P* < 0.05, ^*∗∗*^*P* < 0.01 vs. the control group; ^#^*P* < 0.05, ^##^*P* < 0.01 vs. the model group.

Mice in both the model and APD groups were subjected to intratracheal BLM instillation three days after adaptive feeding. On the eighth day of the experiment, the mice in the APD group received APD via gavage (Figure 1(a)). In comparison to the control group, the body weight of mice in each BLM-instilled group was lower than that of the model group. However, the mice in the APD group exhibited relatively higher body weight than the other groups (Figure 1(b)).

Restrictive ventilatory dysfunction is a crucial parameter associated with the assessment of pulmonary fibrosis, characterized by reduced lung organ adaptability. Cchord serves as the primary indicator of respiratory function in pulmonary fibrosis, representing the change in the slope of lung compliance during lung recovery from a whole lung state to an intrapulmonary pressure equal to atmospheric pressure. While Cchord, IC, FVC, FRC, and TLC were significantly lower in the model group, these values showed significant improvement following APD intervention (*P* < 0.01) (Figures 1(f) and 1(g)). These findings suggest that APD ameliorates restrictive ventilatory dysfunction in mice with pulmonary fibrosis.

On the 28th day, the lung tissue volume in the APD group was relatively higher, and elasticity was restored upon visual observation (Figure 1(c)). The lung index was significantly elevated in mice with pulmonary fibrosis (*P* < 0.01). Notably, after APD treatment, the lung index values appeared to decrease significantly (*P* < 0.05) (Figure 1(d)). These data suggest that APD can attenuate BLM-induced pulmonary fibrosis.

### 3.2. Identification of Active Components in APD Traditional Chinese Medicine Using UHPLC-QE-MS

**Figure 2:**
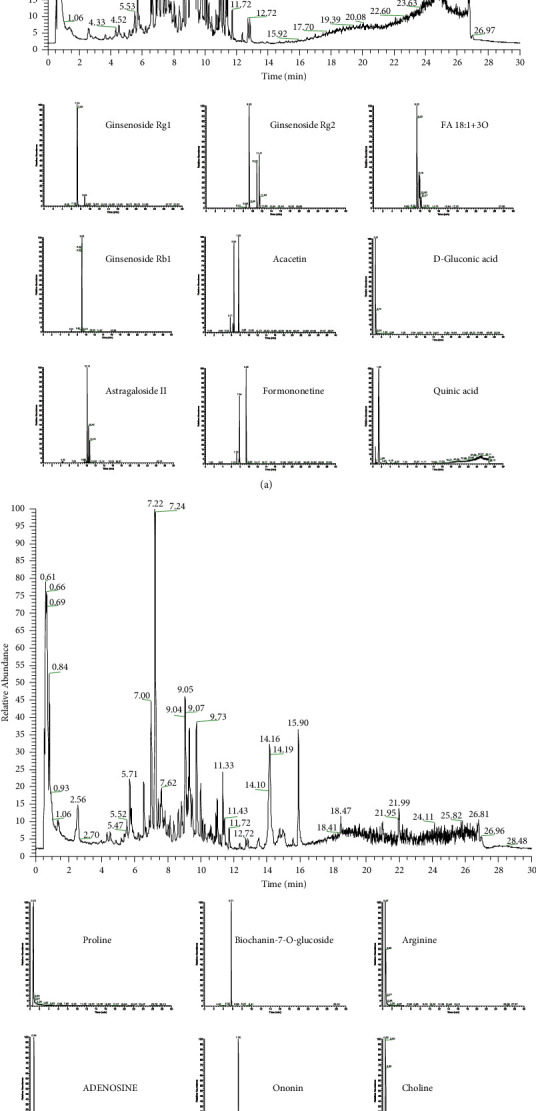
UHPLC-QE-MS analysis of APD: base peak intensity chromatograms in negative and positive modes. (a) UHPLC-QTOF-MS analysis base peak intensity chromatograms of APD in negative mode. (b) UHPLC-QTOF-MS analysis base peak intensity chromatograms of APD in positive mode.

UHPLC-QE-MS was used to identify the active ingredients in APD—a traditional Chinese medicine formulation. The fingerprint of APD was used as the background of UHPLC-QE-MS. The fingerprint of APD had 429 separate main peaks (Figures 2(a) and 2(b)). The relative contents of ginsenoside Rg1, ginsenoside Rg2, FA 18 : 1+3O, ginsenoside Rb1, acacetin, D-gluconic acid, astragaloside II, formononetin, quinic acid, proline, biochanin-7-O-glucoside, arginine, adenosine, ononin, choline, tectochrysin, isoleucine, and glyceryl linolenate were the top nine negative and positive, respectively (Table 2). The primary active ingredient may be composed of one or more of the abovementioned ingredients.

### 3.3. APD Mitigates Lung Tissue Inflammation in Pulmonary Fibrosis Mice

**Figure 3:**
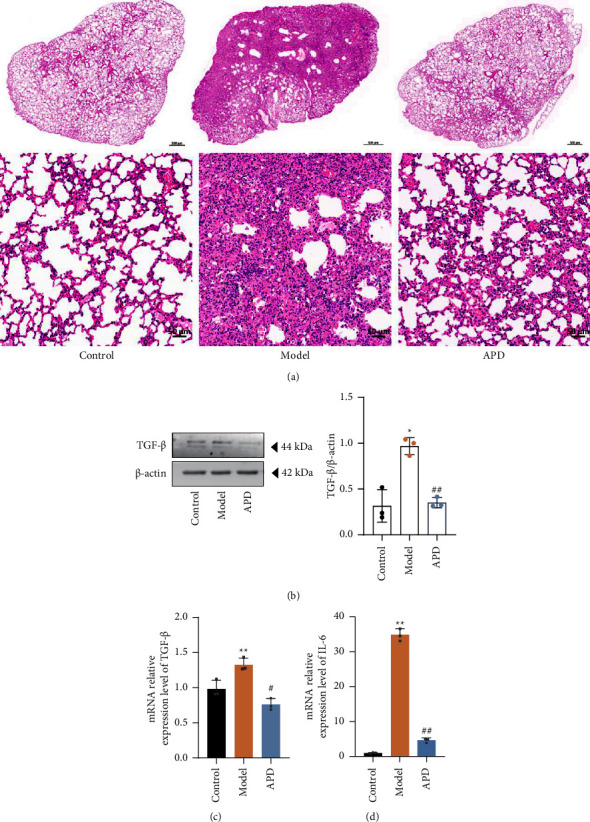
APD effects on lung inflammation and fibrosis: pathological changes and TGF-*β*, IL-6 expression in mice. (a) Pathological changes in lung tissue (hematoxylin and eosin). (b) Lung tissue protein to detect the relative expression of TGF-*β* (*n* = 3). (c) Lung tissue RNA to detect the relative expression of TGF-*β* (*n* = 3). (d) Lung tissue RNA to detect the relative expression of IL-6 (*n* = 3). The data were obtained through three independent experiments. ^*∗*^*P* < 0.05, ^*∗∗*^*P* < 0.01 vs. the control group; ^#^*P* < 0.05, ^##^*P* < 0.01 vs. the model group.

Elevated inflammation is known to be associated with the progression of pulmonary fibrosis [23, 24]. In the current study, the model group exhibited a significant degree of inflammatory infiltration, disrupted alveolar structure, alveolar atrophy, and widening of alveolar septa, while these pathological features were notably reduced in the APD group. The alveolar structure was more complete in the APD group than in the model group (Figure 3(a)). TGF-*β* is an essential inflammatory factor that promotes the transformation of fibroblasts into myofibroblasts to ensure collagen deposition. TGF-*β* levels were significantly higher in the lungs of model mice (*P* < 0.05) and lower in the mice in the APD group (*P* < 0.05) (Figures 3(b) and 3(c)). Epithelial cell-derived interleukin-6 promotes cellular responses in fibrotic diseases [25, 26]. The relative expression of IL-6 mRNA in the model and APD groups was significantly higher and lower than that in the control group, respectively (Figure 3(d)). Collectively, these findings suggest that APD ameliorates lung tissue damage by attenuating inflammatory responses in the mouse model.

### 3.4. APD Attenuates Collagen Deposition in Lung Tissue of Pulmonary Fibrosis Mice

**Figure 4:**
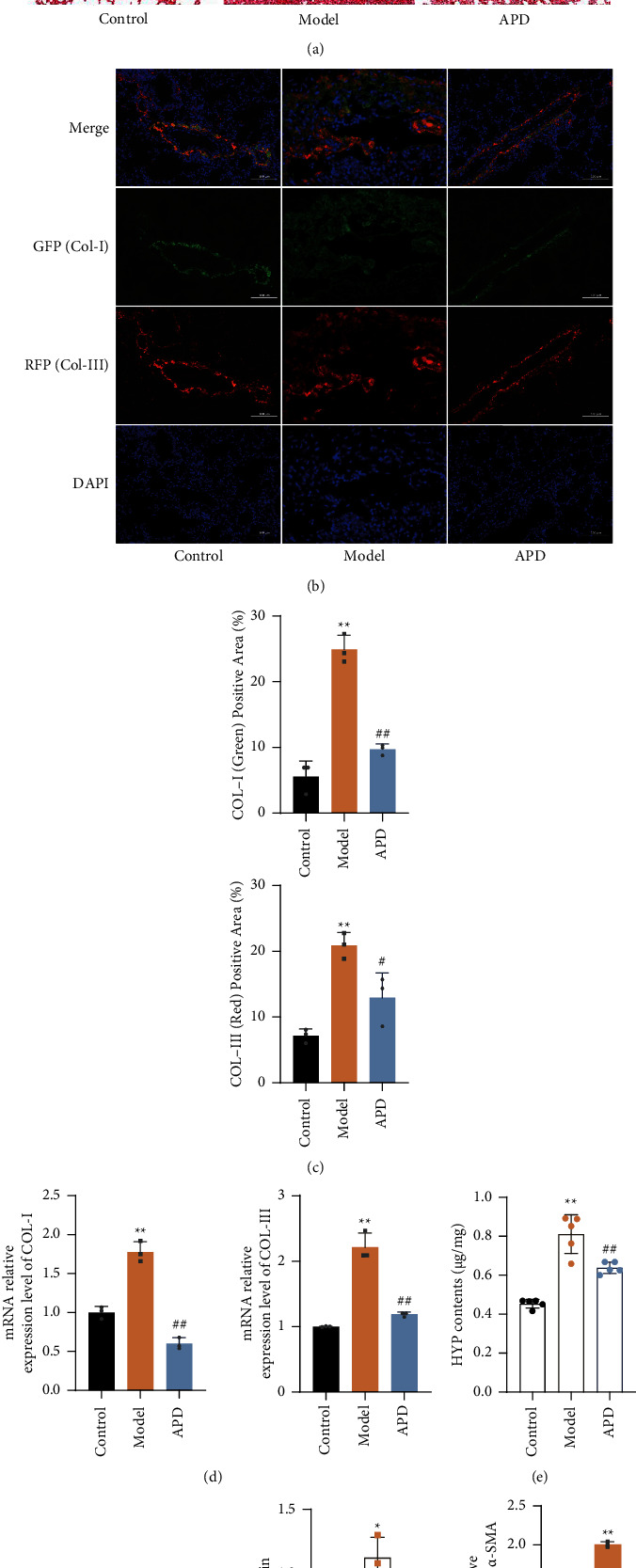
APD reduces collagen deposition and fibrosis markers in pulmonary fibrosis: histological and molecular analyses. (a) Masson staining to compare the collagen deposition in lung tissues of each group. (b) Immunofluorescence to observe COL-I and the expression level of COL-III. (c) Positive area ratio for COL-I (green) and COLI-III (red) using the film of immunofluorescence. (d) RT-qPCR for relative expression of COL-I and COL-III in the lungs (*n* = 3). (e) Determination of the relative content of HYP in lung tissue using an alkaline water hydroxyproline kit (*n* = 5). (f) Lung tissue protein to detect the relative expression of *α*-SMA (*n* = 3). (g) RT-qPCR for relative expression of *α*-SMA in the lungs (*n* = 3).The data were obtained through three independent experiments. ^*∗*^*P* < 0.05, ^*∗∗*^*P* < 0.01 vs. the control group; ^#^*P* < 0.05, ^##^*P* < 0.01 vs. the model group.

Collagen deposition plays a crucial role in pulmonary fibrosis and is key to assessing its severity [27]. For measuring collagen deposition, Masson staining was employed. This revealed structural alterations in lung tissue and significant changes in alveolar septa. The model group exhibited increased light blue collagen deposition around small arteries, bronchi, and in tissue gaps, in contrast to the control group. The APD group, however, demonstrated a marked decrease in cellular collagen deposition compared to the model group (Figure 4(a)). To quantify Col-I and Col-III protein levels in lung tissues, indicators of fibrosis, we utilized immunofluorescence. These proteins were significantly more expressed in the model group than in the control group. Post-APD treatment, a reduction in Col-I and Col-III expression was noted (Figures 4(b)–4(d)). HYP, a specific collagen component in pulmonary fibrosis, was measured using the alkaline hydrolysis method. The model group had significantly higher HYP levels than the control group (*P* < 0.01), which decreased notably following APD treatment (*P* < 0.05) (Figure 4(e)). *α*-SMA, a marker of fibrosis expressed by myofibroblasts, showed higher protein and mRNA levels in the model group than in the control group (*P* < 0.05). In contrast, *α*-SMA levels were reduced in the APD group compared to the model group (*P* < 0.05) (Figures 4(f) and 4(g)). These results indicate the successful establishment of a pulmonary fibrosis model in mice and suggest that APD mitigates pulmonary fibrosis by reducing collagen deposition.

### 3.5. APD Effectively Inhibits Ferroptosis in Pulmonary Fibrosis: A Study in Mouse Lung Tissues

**Figure 5:**
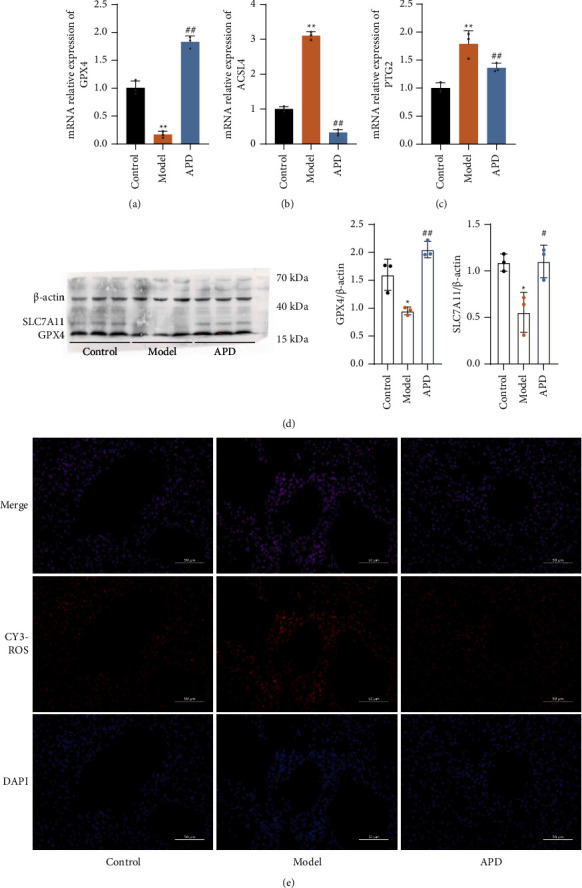
APD mitigates ferroptosis in pulmonary fibrosis: gene expression and oxidative stress markers. (a) RT-qPCR for relative expression of GPX4 in the lungs (*n* = 3). (b) RT-qPCR for the relative expression of ACSL4 in the lungs (*n* = 3). (c) RT-qPCR for the relative expression of PTGS2 in the lungs (*n* = 3). (d) Western blots for protein expression of GPX4 and SLC7A11 in the lungs (*n* = 3). (e) Immunofluorescence staining for ROS. The data were obtained through three independent experiments. ^*∗*^*P* < 0.05, ^*∗∗*^*P* < 0.01 vs. the control group; ^#^*P* < 0.05, ^##^*P* < 0.01 vs. the model group.

APD inhibits activated ferroptosis in the lung tissues of mice with pulmonary fibrosis. Key characteristics of ferroptosis include altered glutathione metabolism, increased lipid peroxidation, and elevated ROS levels. In this study, APD administration significantly reduced ROS levels (Figure 5(e)). The markers GPX4 and SLC7A11, which typically decrease during ferroptosis activation, were found significantly reduced in pulmonary fibrosis mice (*P* < 0.01). APD treatment increased the expression of GPX4 and SLC7A11 in the lung tissues (*P* < 0.01) (Figures 5(a) and 5(d)). The genes PTGS2 and ACSL4, essential for identifying ferroptosis, were upregulated in the model group compared to the control group, as shown by increased ACSL4 and PTGS2 mRNA levels (*P* < 0.01). However, 21 days of APD treatment resulted in a significant decrease in the relative expression of ACSL4 mRNA and PTGS2 mRNA (*P* < 0.01) (Figures 5(b) and 5(c)). These findings indicate that APD may inhibit ferroptosis, thereby mitigating pulmonary fibrosis.

### 3.6. APD's Impact on Ferroptosis and Pulmonary Fibrosis: A Network Pharmacology Study on Gene Interactions

**Figure 6:**
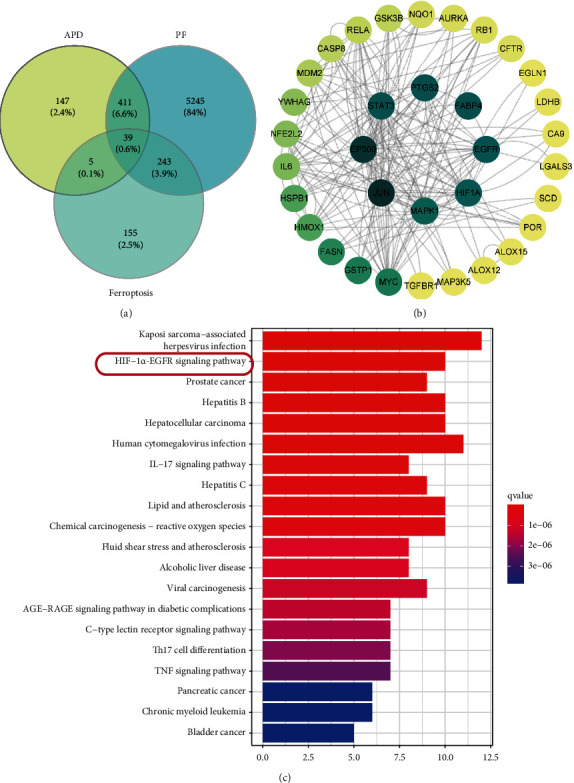
Exploring APD's mechanism on ferroptosis and pulmonary fibrosis: network pharmacology insights. (a) Venn diagram analysis of the common target genes of APD (*Astragalus* and *Panax notoginseng*), ferroptosis, and pulmonary fibrosis. (b) Protein-protein interactions of the common targets of APD (*Astragalus* and *Panax notoginseng*), ferroptosis, and pulmonary fibrosis. (c) Kyoto Encyclopedia of Genes and Genomes analysis of the common targets of APD (*Astragalus* and *Panax notoginseng*), ferroptosis, and pulmonary fibrosis.

Analyzing the impact of APD on genes is associated with ferroptosis and pulmonary fibrosis through network pharmacology. To explore the intersection of targets related to APD, ferroptosis, and pulmonary fibrosis, we identified 602 genes linked to the pharmacological effects of Astragalus and Panax notoginseng from the TCMSP database, extracted 5938 genes associated with pulmonary fibrosis based on the DisGeNET, GeneCards, and OMIM databases, and pinpointed 442 genes connected to ferroptosis from the same sources. Venn diagram analysis showed 39 shared gene targets (Figure 6(a)). Protein-protein interaction network analysis highlighted that among these 39 genes, JUN, EP300, and STAT3 were notably interconnected (Figure 6(b)). Further analysis using the Kyoto Encyclopedia of Genes and Genomes suggested a crucial role for the Hif-1*α*-EGFR signaling pathway in ferroptosis and pulmonary fibrosis (Figure 6(c)). Therefore, we hypothesize that the Hif-1*α*-EGFR signaling pathway may be a potential therapeutic target for APD.

### 3.7. The Role of Hif-1*α*-EGFR in APD-Regulated Ferroptosis Alleviating Pulmonary Fibrosis

**Figure 7:**
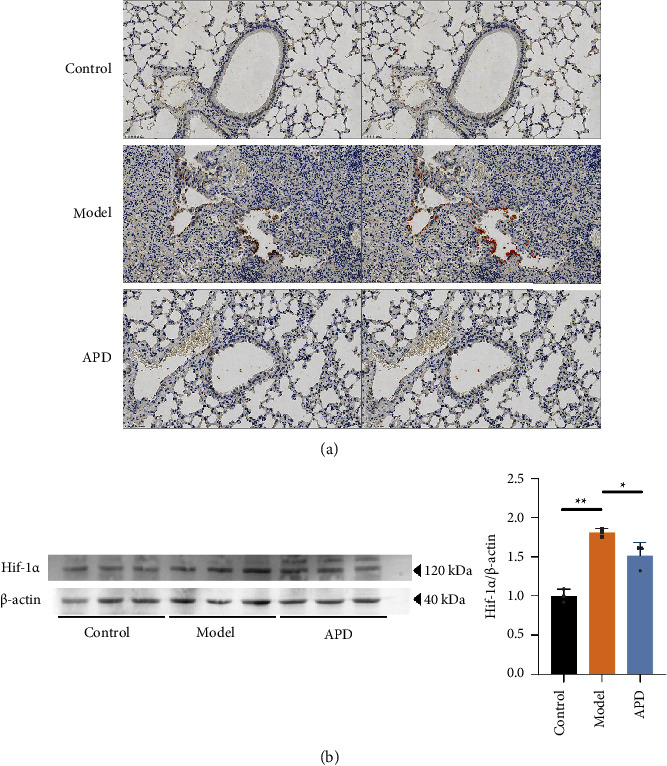
APD modulates Hif-1*α*-EGFR signaling in ferroptosis and pulmonary fibrosis: evidence from immunohistochemistry and western blot analysis. (a) Immunohistochemistry for EGFR in the lungs (the red area, on the right, is a positive mark after enhancement). (b) Western blots for Hif-1*α* (*n* = 3). The data were obtained through three independent experiments. ^*∗*^*P* < 0.05, ^*∗∗*^*P* < 0.01 vs. the control group; ^#^*P* < 0.05, ^##^*P* < 0.01 vs. the model group.

Given our findings, the Hif-1*α*-EGFR signaling pathway appears to be crucial for APD's regulation of ferroptosis in pulmonary fibrosis. We therefore examined its activity in pulmonary fibrosis cases. Immunohistochemistry showed distinct brown deposits, indicative of EGFR protein presence, in the tracheal epithelium of pulmonary fibrosis mice. APD treatment significantly diminished this staining (Figure 7(a)), indicating a reduction in Hif-1*α* protein levels, which were elevated in the model group (Figure 7(b)). These results suggest that inhibiting the Hif-1*α*-EGFR pathway may be essential in the APD-mediated regulation of ferroptosis and the mitigation of pulmonary fibrosis.

## 4. Discussion

Pulmonary fibrosis, commonly seen in the advanced stages of various respiratory diseases, is characterized by a progressive exacerbation of specific pulmonary pathologies [28]. Current treatment protocols, including antifibrotic agents, antioxidant therapy, anti-inflammatory drugs, and lung transplantation, face challenges due to adverse effects and limited efficacy [29]. This highlights the urgent need to develop new drugs to improve or prevent the progression of pulmonary fibrosis to respiratory failure.

Inflammation and collagen deposition are pivotal in pulmonary fibrosis. The inflammatory response involves diverse immune cells and mediators, which initiate and worsen fibrosis, leading to tissue remodeling and damage [30, 31]. Excessive collagen accumulation in the extracellular matrix disrupts lung structure and function [32, 33]. Targeting these processes is crucial for therapeutic intervention.

Numerous studies have demonstrated the efficacy of plant-based treatments in combating pulmonary fibrosis. *Arenaria kansuensis* significantly reduced inflammatory infiltration, collagen deposition, and oxidative stress in a paraquat-induced pulmonary fibrosis animal model [34]. Bergenin showed protective effects in a bleomycin-induced mouse model [35]. Osthole exhibited antioxidative stress effects in lung fibroblasts [36]. Thus, exploring plant-based treatments for pulmonary fibrosis is promising.

Astragalus and Panax notoginseng, commonly used in traditional medicine for Qi deficiency and blood stasis, have shown pharmacological effects on various systems, including respiratory, immune, central nervous, cardiovascular, endocrine systems, and cancer [37–40]. Diao Hui et al. demonstrated the anti-inflammatory effects of APD and its protection against acute kidney injury [41]. Astragalus and Panax notoginseng regulate multiple targets, pertinent to the varied symptoms of pulmonary fibrosis, such as restrictive ventilation dysfunction, inflammation, and collagen deposition [24, 42, 43]. In our animal experiments, APD improved ventilation dysfunction, reduced inflammation, and decreased collagen deposition, suggesting its potential in preventing pulmonary fibrosis. The multifaceted pharmacological actions of APD align with the complex pathogenesis of diseases, making it a promising candidate for treating pulmonary fibrosis.

Ferroptosis, a recently identified iron-dependent form of cell death, results from increased ROS and lipid peroxidation. This regulatory cell death process is essential for maintaining cellular morphological and functional homeostasis. Inhibition of ferroptosis has been identified as a key factor in fibrotic diseases, such as myocardial and liver fibrosis [44, 45]. Consistent with these findings, our study on bleomycin-induced pulmonary fibrosis in mice revealed increased levels of ACSL4 and PTGS2, which promote oxidative stress, and decreased expression of GPX4 and SLC7A11, inhibitors of oxidative stress. These results suggest the occurrence of ferroptosis in pulmonary fibrosis, aligning with prior studies [46, 47]. Interestingly, APD downregulates ACSL4, PTGS2, and ROS while upregulating GPX4 and SLC7A11, indicating its role in inhibiting ferroptosis in pulmonary fibrosis. Thus, APD may alleviate pulmonary fibrosis by inhibiting ferroptosis, providing a potential targeted therapy approach.

Hif-1 is a heterodimer comprising two basic helix-loop-helix Per-ARNT-Sim proteins, Hif-1*α* and Hif-1*β*. Hif-1*α* is primarily expressed under hypoxic conditions, while Hif-1*β* is constitutively expressed. Hif-1*α* plays a crucial role in the hypoxic response of tumor cells, controlling the upregulation of vital factors like VEGF, necessary for solid tumor expansion. Conversely, Hif-1*β* functions as an aromatic hydrocarbon receptor nuclear transporter and is key to various biological responses [48–50]. Recent studies have identified a link between Hif-1*α* and ferroptosis in other diseases [51]. In this study, we explored the roles of Hif-1*α* and its downstream molecule EGFR in pulmonary fibrosis mice and the APD group, suggesting that APD might regulate ferroptosis by inhibiting the Hif-1*α*-EGFR pathway, thereby alleviating pulmonary fibrosis (Figures 7(a) and 7(b)).

While our results indicate APD's antifibrotic effects in pulmonary fibrosis, further research is required. Specifically, the mechanism involving the inhibition of Hif-1*α*-EGFR and its relation to ferroptosis in pulmonary fibrosis needs more exploration. Last, a significant limitation of this study is the lack of supporting clinical data.

## 5. Conclusion

Our findings suggest that APDs, with their antifibrotic properties, represent a viable treatment option for pulmonary fibrosis. These agents function by inhibiting ferroptosis through the activation of the Hif-1*α*-EGFR signaling pathway. This leads to a reduction in collagen deposition and an alleviation of restrictive ventilatory dysfunction, both of which are characteristic features of pulmonary fibrosis.

## Figures and Tables

**Table 1:** Mice primer pairs used for RT-qPCR analysis.

Gene	Forward primer (5′⟶3′)	Reverse primer (5′⟶3′)
TGF-*β*1	CACAAGAGCAGTGAGCGCTGAA	TGATACGCCTGAGTGGCTGTCT
*α*-SMA	CGGCAGTAGTCACGAAGGAATAGC	TGCTGGACTCTGGAGATGGTGTG
PTGS2	GCGACATACTCAAGCAGGAGCA	AGTGGTAACCGCTCAGGTGTTG
ACSL4	GCTATCTCCTCAGACACACCGA	AGGTGCTCCAACTCTGCCAGTA
Gpx4	ACAAGAACGGCTGCGTGGTGAA	GCCACACACTTGTGGAGCTAGA
Nrf2	TCTTGGAGTAAGTCGAGAAGTGT	GTTGAAACTGAGCGAAAAAGGC
HSF1	GCACACTCTGTGCCCAAGTATG	AGCTGGTGACAGCATCAGAGGA
Hspb1	GCTCACAGTGAAGACCAAGGAAG	TGAAGCACCGAGAGATGTAGCC
Keap1	ATCCAGAGAGGAATGAGTGGCG	TCAACTGGTCCTGCCCATCGTA
Gapdh	CATCACTGCCACCCAGAAGACTG	ATGCCAGTGAGCTTCCCGTTCAG

**Table 2:** Identification of components of APD using UHPLC-QTOF-MS.

	NameEN	Composite score	Name	Class	mzmed		rtmed	Peak area	Electric
1	Ginsenoside Rg1	0.915318462	M845.494T434.801	Terpenoids	845.4935802		434.8015	3488809931	−H
2	Ginsenoside Rg2	0.708125308	M829.495T555.211	Terpenoids	829.4947253		555.211	1523762424	−H
3	FA 18:1+3O	0.947362692	M329.233T559.064	Miscellaneous	329.2331244		559.064	1454218946	−H
4	Ginsenoside Rb1	0.892871308	M1107.593T543.773	Terpenoids	1107.593148		543.773	966036633.3	−H
5	Acacetin	0.994974692	M283.062T393.424	Flavonoids		283.0618205	393.424	939086813.1	−H
6	D-gluconic acid	0.957612231	M195.051T39.469	Organic acids and derivatives		195.0512625	39.4687	893006112.9	−H
7	Astragaloside II	0.962389308	M871.469T606.642	Terpenoids		871.4692999	606.642	891095687	−H
8	Formononetin	0.993590154	M267.066T563.276	Flavonoids		267.0662804	563.276	682296537	−H
9	Quinic acid	0.602413077	M191.056T82.676	Organooxygen compounds		191.0560524	82.6757	376872893.8	−H
10	Proline	1	M116.070T42.358	Alkaloids		116.0703625	42.35815	3753378731	+H
11	Biochanin-7-O-glucoside	0.909076692	M447.128T343.159	Flavonoids		447.1282951	343.159	2070895117	+H
12	Arginine	0.985448385	M175.119T38.285	Amino acid derivatives		175.1185922	38.2845	2004833077	+H
13	ADENOSINE	0.842052692	M268.104T50.335	Alkaloids		268.1038796	50.3354	1506453675	+H
14	Ononin	0.941818	M431.132T432.306	Flavonoids		431.1320572	432.306	1265507512	+H
15	Choline	0.983240692	M104.107T36.490	Organonitrogen compounds		104.1067169	36.4899	1189143338	+H
16	Tectochrysin	0.974453769	M269.080T563.236	Flavonoids		269.08006	563.236	1074176459	+H
17	Isoleucine	0.956832231	M132.102T56.568	Amino acid derivatives		132.1018909	56.5683	800831223.6	+H
18	Glyceryl linolenate	1	M375.250T849.468	Miscellaneous		375.2498279	849.468	620348177.8	+H

## Data Availability

The study data generated in the present study are included in the figures and/or tables of this article. The network pharmacology data generated in the present study may be found in the corresponding URL that you find in the material methodology.
